# New Multi-Step Iterative Methods for Solving Systems of Nonlinear Equations and Their Application on GNSS Pseudorange Equations

**DOI:** 10.3390/s20215976

**Published:** 2020-10-22

**Authors:** Kalyanasundaram Madhu, Arul Elango, René Jr Landry, Mo’tassem Al-arydah

**Affiliations:** 1 Department of Mathematics, Khalifa University, Abu Dhabi P.O. Box 127788, UAE; kalyanasundaram.mathu@ku.ac.ae (K.M.); motassem.alarydah@ku.ac.ae (M.A.-a); 2 LASSENA Laboratory, Department of Electrical Engineering, Ecole de Technologie Superieure, Montréal, QC H3C 1K3, Canada; renejr.landry@etsmtl.ca

**Keywords:** navigation equations, higher-order, convergence order, multi-step method, GNSS, GDOP, 65H05

## Abstract

A two-step fifth and a multi-step 5+3r order iterative method are derived, r≥1 for finding the solution of system of nonlinear equations. The new two-step fifth order method requires two functions, two first order derivatives, and the multi-step methods needs a additional function per step. The performance of this method has been tested with finding solutions to several test problems then applied to solving pseudorange nonlinear equations on Global Navigation Satellite Signal (GNSS). To solve the problem, at least four satellite’s measurements are needed to locate the user position and receiver time offset. In this work, a number of satellites from 4 to 8 are considered such that the number of equations is more than the number of unknown variables to calculate the user position. Moreover, the Geometrical Dilution of Precision (GDOP) values are computed based on the satellite selection algorithm (fuzzy logic method) which could be able to bring the best suitable combination of satellites. We have restricted the number of satellites to 4 to 6 for solving the pseudorange equations to get better GDOP value even after increasing the number of satellites beyond six also yields a 0.4075 GDOP value. Actually, the conventional methods utilized in the position calculation module of the GNSS receiver typically converge with six iterations for finding the user position whereas the proposed method takes only three iterations which really decreases the computation time which provide quicker position calculation. A practical study was done to evaluate the computation efficiency index (CE) and efficiency index (IE) of the new model. From the simulation outcomes, it has been noted that the new method is more efficient and converges 33% faster than the conventional iterative methods with good accuracy of 92%.

## 1. Introduction

Numerical analysis is a comprehensive subject which is interconnected with applied mathematics, various fields of science and engineering, medical, etc. The most elemental and primal problem in this subject is to find the efficient and precise approximate solution $x^{\left(*\right)}\in D$ of a systems of nonlinear F(x)=0, where $F:D\subset R^{n}\to R^{n}$, and this type of problem will be solved by the famous Newton’s method ($2^{nd}NR$) which has second order convergence [1] by

(1) $$y\left(x^{\left(n\right)}\right)=x^{\left(n\right)}-{\left[F^{\prime }\left(x^{\left(n\right)}\right)\right]}^{-1}F\left(x^{\left(n\right)}\right),\;n=0,1,2,...$$

where $F^{\prime }\left(x^{\left(n\right)}\right)$ is the Jacobian matrix of the function and requires the evaluation of one *F*, one $F^{\prime }$ per iteration. Traub [2] gave suggestion a multi-point schemes are best one to increase the order of convergence when absent of second derivatives, such methods have given in the literature; see [3,4,5,6,7]. Traub [2] gave a double-step Newton’s type method ($3^{rd}TM$) having convergence order 3 by two *F*, one $F^{\prime }$ evaluations

(2) $$z\left(x^{\left(n\right)}\right)=y\left(x^{\left(n\right)}\right)-\left[F^{\prime }\left(x^{\left(n\right)}\right)\right){]}^{-1}F\left(y\left(x^{\left(n\right)}\right)\right),$$

where $y(x^{\left(n\right)})$ is given in Equation (1). The two-step Newton’s method ($4^{th}NR$) with convergence order four

(3) $$x^{\left(n+1\right)}=y\left(x^{\left(n\right)}\right)-{\left[F^{\prime }\left(y\left(x^{\left(n\right)}\right)\right)\right]}^{-1}F\left(y\left(x^{\left(n\right)}\right)\right),$$

was reconstructed by Noor et al. [6], where *F* and $F^{\prime }$ are evaluated two times each. Abad et al. [3] suggested the Newton type methods to get a 3-step 4th order method ($4^{th}ACT$), where two evaluations of *F*, two $F^{\prime }$ are used

(4) $$x^{\left(n+1\right)}=y\left(x^{\left(n\right)}\right)-{\left[F^{\prime }\left(z\left(x^{\left(n\right)}\right)\right)\right]}^{-1}F\left(y\left(x^{\left(n\right)}\right)\right).$$

where $z(x^{\left(n\right)})$ is given in Equation (2). Sharma et al. [7] presented a two-step 4th order method ($4^{th}SGS$), where 1 time *F* and 2 times $F^{\prime }$ are evaluated per cycle, and it is given here

(5) $$\begin{array}{cc} \; & x^{\left(n+1\right)}=x^{\left(n\right)}-\left(-\frac{1}{2}I+\frac{9}{8}{\left[F^{\prime }\left(y_{1}\left(x^{\left(n\right)}\right)\right)\right]}^{-1}F^{\prime }\left(x^{\left(n\right)}\right)+\frac{3}{8}{\left[F^{\prime }\left(x^{\left(n\right)}\right)\right]}^{-1}F^{\prime }\left(y_{1}\left(x^{\left(n\right)}\right)\right)\right)u\left(x^{\left(n\right)}\right),\\ \; & y_{1}\left(x^{\left(n\right)}\right)=x^{\left(n\right)}-\frac{2}{3}u\left(x^{\left(n\right)}\right),u\left(x^{\left(n\right)}\right)={\left[F^{\prime }\left(x^{\left(n\right)}\right)\right]}^{-1}F\left(x^{\left(n\right)}\right). \end{array}$$

Babajee et al. [8] gave a two-step 4th order scheme ($4^{th}BCST$), where 1 time *F*, 2 time $F^{\prime }$ are evaluated per cycle and its given below

(6) $$\begin{array}{cc} \; & x^{\left(n+1\right)}=x^{\left(n\right)}-W\left(x^{\left(n\right)}\right){\left(\frac{1}{2}\left(F^{\prime }\left(x^{\left(n\right)}\right)+F^{\prime }\left(y\left(x^{\left(n\right)}\right)\right)\right)\right)}^{-1}F\left(x^{\left(n\right)}\right),\\ W(x^{\left(n\right)}) & =I-\frac{1}{4}\left(\tau \left(x^{\left(n\right)}\right)-I\right)+\frac{3}{4}{\left(\tau \left(x^{\left(n\right)}\right)-I\right)}^{2},\;\tau \left(x^{\left(n\right)}\right)=F^{\prime }{\left(x^{\left(n\right)}\right)}^{-1}F^{\prime }\left(y\left(x^{\left(n\right)}\right)\right). \end{array}$$

Abad et al. [3], a different combination was used to get a three-step fifth order method, where three functions, two Jacobian matrices and their inverses were evaluated, and it is given below

(7) $$x^{\left(n+1\right)}=z\left(x^{\left(n\right)}\right)-{\left[F^{\prime }\left(y\left(x^{\left(n\right)}\right)\right)\right]}^{-1}F\left(z\left(x^{\left(n\right)}\right)\right).$$

Madhu et al. [9] improved double step Newton’s method and also developed its multi-step version are given below

(8) $$\begin{array}{cc} \; & x^{\left(n+1\right)}=y\left(x^{\left(n\right)}\right)-H_{1}\left(x^{\left(n\right)}\right){\left[F^{\prime }\left(x^{\left(n\right)}\right)\right]}^{-1}F\left(y\left(x^{\left(n\right)}\right)\right),\\ \; & H_{1}\left(x^{\left(n\right)}\right)=2I-\tau \left(x^{\left(n\right)}\right)+\frac{5}{4}{\left(\tau \left(x^{\left(n\right)}\right)-I\right)}^{2},\;\tau \left(x^{\left(n\right)}\right)={\left[F^{\prime }\left(x^{\left(n\right)}\right)\right]}^{-1}F^{\prime }\left(y\left(x^{\left(n\right)}\right)\right). \end{array}$$

### Literature Survey

Yang [10] implemented an algebraic high compatible positioning via non iterative method employing direct solution of the double-difference pseudorange equations for solving the GPS double-difference pseudorange equations if two or more GPS receivers operate simultaneously. Pachter et al. [11] improved the performance of estimation under high geometric dilution of precision (GDOP) conditions. In this work, the stochastic modeling algorithm for the GPS pseudorange equations is derived and compared with the conventional ILS algorithm. In order to achieve optimality when the trilateration system of equations becomes over-determined, Li et al. [12] proposed an algorithm which uses the direct linearization technique to reduce the computation time overhead. Similarly, to investigate land-based radio positioning, Kwang-Soob et al. [13], mathematically derived two-dimensional positioning based on GPS Pseudorange linearized state equation in which the geometry model with respect to triangles are formed using unit-vectors. GPS navigation solution using iterative least absolute deviation approach has been described by Jwoa et al. [14] based on the Least Absolute Deviation (LAD) criterion for estimating navigation solutions since the least square technique is very much sensitive to accuracy and the performance for mitigating GPS multipath errors. In Awange et al. [15], an alternative closed form GPS pseudo-ranging four-point problem P4P in matrix form using multi polynomial resultant and Groebner basis has been implemented in algebraic software such as Mathematica and Maple to solve the nonlinear GPS pseudo-ranging four-point equations. In general, the positioning module of the GNSS receiver use conventional Taylor series and Bancroft algorithms for solving non-linear pseudorange equations. Elnaggar [16] presented a modified Taylor series method which linearizes the pseudorange equation by considering four satellite coordinates and pseudorange values for improving the positioning. On the other hand, Abad et al. [3] described a solution for GPS pseudorange equations by solving different iterative methods for four visible satellites.

**Figure 1:**
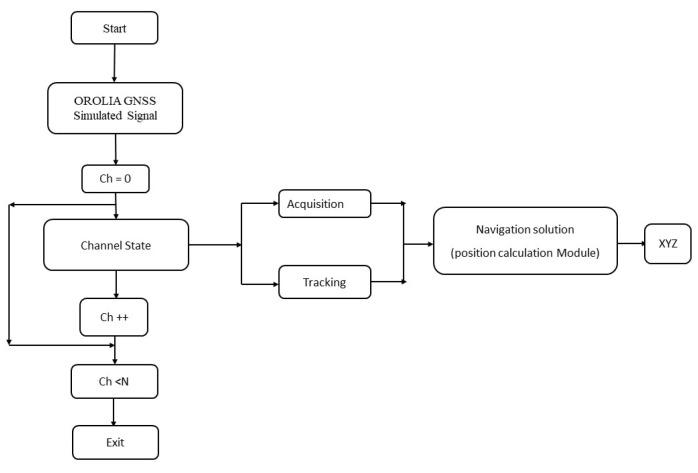
Flowchart of GNSS of positioning.

In this work, we have considered four or more than four visible satellites scenario for linearizing the pseudorange equations for obtaining better GDOP value and one can notice that the computational order calculation is not matching with the theoretical order when we increased the number of satellites more than four, most of the iterative methods does not match with the theoretical order but the new multistep iterative method described here approximately matches with the order and still preserves the accuracy. Initially the set of nonlinear problems are solved using various iterative methods and then the GNSS pseudorange nonlinear equations are solved in the next section. The input GNSS signal simulated from OROLIA simulator has been given as an input to the software based GNSS receiver to find the user position. The proposed iterative technique used in the position calculation module of the GNSS receiver is depicted in Figure 1.

The input signal is acquired and the number of visible satellites are passed on to the tracking module to lock the code and carrier phase. Further, in the position calculation module the iterative methods are used to find the user position. The conventional way of solving GPS pseudorange equations involve Taylor series and iterative methods which typically take more than 6 iterations if the number of visible satellites are considered to be 4 but in the proposed work, the user position is computed within 3 iterations that significantly reduce the computation time in the position calculation module of the GPS receiver.

The content of the paper is formulated as follows: a new method of 5th order and their multi-step scheme with 5+3r order, and its convergence analysis are presented in Section 2. In Section 3, numerical test problems are given and it is theoretical convergence order is analyzed and the results are validated through Matlab simulation. The efficiency index and computational efficiency index are given in Section 4. The GNSS application problem is carried out for new methods and few existing methods are described in Section 5. Test problems and application problem results are discussed in Section 6. The final section is concluded with extending this work on solving position calculation of GNSS multi-constellation receiver.

## 2. Mathematical Description of New Method and Its Convergence Analysis

### 2.1. A Two-Step Fifth-Order Method

We present here a new fifth order method ($5^{th}PM$), derived from combining of Traub’s method (2) and weight functions. It is given as follows

(9) $$\begin{array}{ccc} x^{\left(n+1\right)} & = & y\left(x^{\left(n\right)}\right)-H_{1}\left(x^{\left(n\right)}\right){\left[F^{\prime }\left(x^{\left(n\right)}\right)\right]}^{-1}F\left(y\left(x^{\left(n\right)}\right)\right),\\ H_{1}\left(x^{\left(n\right)}\right) & = & \tau \left(x^{\left(n\right)}\right)+\frac{1}{4}{\left(\tau \left(x^{\left(n\right)}\right)-I\right)}^{2},\;\;\mathrm{where}\;\;\tau \left(x^{\left(n\right)}\right)={\left[F^{\prime }\left(y\left(x^{\left(n\right)}\right)\right)\right]}^{-1}F^{\prime }\left(x^{\left(n\right)}\right), \end{array}$$

$y(x^{\left(n\right)})$ is given in Equation (1), and *I* is the identity matrix by n×n.

### 2.2. A Multi-Step ${\left(5+3r\right)}^{th}$-Order Method

Further, we improved the 5th order method to 5+3r order method by additional one function per each step. It is shown below

(10) $$\begin{array}{cc} \; & x^{\left(n+1\right)}={\left(5+3r\right)}^{th}PM\left(x^{\left(n\right)}\right)=\psi_{r}\left(x^{\left(n\right)}\right),\\ \; & \psi_{m}\left(x^{\left(n\right)}\right)=\psi_{m-1}\left(x^{\left(n\right)}\right)-H_{2}\left(x^{\left(n\right)}\right){\left[F^{\prime }\left(x^{\left(n\right)}\right)\right]}^{-1}F\left(\psi_{m-1}\left(x^{\left(n\right)}\right)\right),\\ \; & H_{2}\left(x^{\left(n\right)}\right)=\tau \left(x^{\left(n\right)}\right)+\frac{1}{2}{\left(\tau \left(x^{\left(n\right)}\right)-I\right)}^{2},\\ \; & \psi_{0}\left(x^{\left(n\right)}\right)=5^{th}PM\left(x^{\left(n\right)}\right),\;m=1\left(1\right)r. \end{array}$$

Note that, the spacial case r=0 is the $5^{th}PM$ method is given in (9). It is noted that the $5^{th}PM$ is Cordero et al. [17] they used three arbitrary parameters for obtaining the method. However, we have used the Taylor series technique to propose the $5^{th}PM$ method (9). We can say that the method $5^{th}PM$ is reconstructed of Cordero et al. [17] method.

### 2.3. Convergence Analysis

**Theorem** **1.** Let F be sufficiently differentiable in an open convex set D, $x^{\left(*\right)}\in D$, the roots of the problem F(x)=0. $F^{\prime }\left(x\right)$ is continuing function and nonsingular in $x^{\left(*\right)}$. Then the method (9) converging to the $x^{\left(*\right)}$ with convergence order at least five,

(11) $$\begin{array}{cc} \epsilon^{\left(n+1\right)} & =5^{th}PM\left(x^{\left(n\right)}\right)-x^{\left(*\right)}=M_{1}{\epsilon^{\left(n\right)}}^{5}+O\left({\epsilon^{\left(n\right)}}^{6}\right),\;\epsilon^{\left(n\right)}=x^{\left(n\right)}-x^{\left(*\right)}\\ M_{1} & =2C_{2}^{4}-C_{2}C_{3}C_{2},\;where\;C_{h}=\left(1/h!\right){\left[F^{\prime }\left(x^{\left(*\right)}\right)\right]}^{-1}F^{\left(h\right)}\left(x^{\left(*\right)}\right),h\;\geq 2\left(1\right)n. \end{array}$$

**Proof.**  Expanding *F* and $F^{\prime }$ around $x^{\left(*\right)}$ at the point $x^{\left(n\right)}$, we obtain

(12) $$F\left(x^{\left(n\right)}\right)=F^{\prime }\left(x^{\left(*\right)}\right)\left[\epsilon^{\left(n\right)}+C_{2}{\epsilon^{\left(n\right)}}^{2}+C_{3}{\epsilon^{\left(n\right)}}^{3}+C_{4}{\epsilon^{\left(n\right)}}^{4}+C_{5}{\epsilon^{\left(n\right)}}^{5}\right]+O\left({\epsilon^{\left(n\right)}}^{6}\right),$$

and

(13) $$F^{\prime }\left(x^{\left(n\right)}\right)=F^{\prime }\left(x^{\left(*\right)}\right)\left[I+2C_{2}\epsilon^{\left(n\right)}+3C_{3}{\epsilon^{\left(n\right)}}^{2}+4C_{4}{\epsilon^{\left(n\right)}}^{3}+5C_{5}{\epsilon^{\left(n\right)}}^{4}\right]+O\left({\epsilon^{\left(n\right)}}^{5}\right).$$

We have

(14) $${\left[F^{\prime }\left(x^{\left(n\right)}\right)\right]}^{-1}={\left[F^{\prime }\left(x^{\left(*\right)}\right)\right]}^{-1}\left[I+X_{1}\epsilon^{\left(n\right)}+X_{2}{\epsilon^{\left(n\right)}}^{2}+X_{3}{\epsilon^{\left(n\right)}}^{3}+X_{4}{\epsilon^{\left(n\right)}}^{4}\right]+O\left({\epsilon^{\left(n\right)}}^{5}\right),$$

here $X_{1}=-2C_{2}$, $X_{2}=-3C_{3}+4C_{2}^{2}$, $X_{3}=+6C_{2}C_{3}-8C_{2}^{3}-4C_{4}+6C_{3}C_{2}$ and $X_{4}=-5C_{5}+9C_{3}^{2}+8C_{2}C_{4}+8C_{4}C_{2}+16C_{2}^{4}-12C_{2}^{2}C_{3}-12C_{3}C_{2}^{2}-12C_{2}C_{3}C_{2}$. Then

(15) $$\begin{array}{cc} \; & {\left[F^{\prime }\left(x^{\left(n\right)}\right)\right]}^{-1}F\left(x^{\left(n\right)}\right)=\epsilon^{\left(n\right)}-C_{2}{\epsilon^{\left(n\right)}}^{2}+\left(2C_{2}^{2}-2C_{3}\right){\epsilon^{\left(n\right)}}^{3}+\left(-3C_{4}-4C_{2}^{3}+4C_{2}C_{3}+3C_{3}C_{2}\right){\epsilon^{\left(n\right)}}^{4}\\ \; & +\left(6C_{3}^{2}+8C_{2}^{4}-8C_{2}^{2}C_{3}-6C_{2}C_{3}C_{2}-6C_{3}C_{2}^{2}+6C_{2}C_{4}+4C_{4}C_{2}-4C_{5}\right){\epsilon^{\left(n\right)}}^{5}+O\left({\epsilon^{\left(n\right)}}^{6}\right). \end{array}$$

In addition, we have

(16) $$\begin{array}{cc} y(x^{\left(n\right)}) & =x^{\left(*\right)}+C_{2}{\epsilon^{\left(n\right)}}^{2}+\left(-2C_{2}^{2}+2C_{3}\right){\epsilon^{\left(n\right)}}^{3}+\left(3C_{4}-4C_{2}C_{3}+4C_{2}^{3}-3C_{3}C_{2}\right){\epsilon^{\left(n\right)}}^{4}\\ \; & +\left(-6C_{3}^{2}-8C_{2}^{4}+8C_{2}^{2}C_{3}+6C_{2}C_{3}C_{2}+6C_{3}C_{2}^{2}-6C_{2}C_{4}-4C_{4}C_{2}+4C_{5}\right){\epsilon^{\left(n\right)}}^{5}. \end{array}$$

Expanding *F* and $F^{\prime }$ around $x^{\left(*\right)}$ at $y(x^{\left(n\right)})$ in Taylor series respectively given below

(17) $$\begin{array}{ccc} F(y\left(x^{\left(n\right)}\right)) & = & F^{\prime }\left(x^{\left(*\right)}\right)\left[\left(y\left(x^{\left(n\right)}\right)-x^{\left(*\right)}\right)+C_{2}{\left(y\left(x^{\left(n\right)}\right)-x^{\left(*\right)}\right)}^{2}+C_{3}{\left(y\left(x^{\left(n\right)}\right)-x^{\left(*\right)}\right)}^{3}+...\right]\\ & = & F^{\prime }\left(x^{\left(*\right)}\right)[C_{2}{\epsilon^{\left(n\right)}}^{2}+2\left(-C_{2}^{2}+C_{3}\right){\epsilon^{\left(n\right)}}^{3}+\left(3C_{4}+5C_{2}^{3}-4C_{2}C_{3}-3C_{3}C_{2}\right){\epsilon^{\left(n\right)}}^{4}\\ & & +\left(-6C_{3}^{2}-12C_{2}^{4}+10C_{2}^{2}C_{3}+8C_{2}C_{3}C_{2}+6C_{3}C_{2}^{2}-6C_{2}C_{4}-4C_{4}C_{2}+4C_{5}\right){\epsilon^{\left(n\right)}}^{5}], \end{array}$$

(18) $$\begin{array}{cc} & F^{\prime }\left(y\left(x^{\left(n\right)}\right)\right)=F^{\prime }\left(x^{\left(*\right)}\right)\left[I+P_{1}{\epsilon^{\left(n\right)}}^{2}+P_{2}{\epsilon^{\left(n\right)}}^{3}+P_{3}{\epsilon^{\left(n\right)}}^{4}\right]+O\left({\epsilon^{\left(n\right)}}^{5}\right), \end{array}$$

where

$$P_{1}=2C_{2}^{2},P_{2}=4C_{2}C_{3}-4C_{2}^{3},P_{3}=8C_{2}^{4}+6C_{2}C_{4}-8C_{2}^{2}C_{3}+3C_{3}C_{2}^{2}-6C_{2}C_{3}C_{2}.$$

Using (14) and (18), we have

(19) $$\begin{array}{cc} \; & {\left[F^{\prime }\left(y\left(x^{\left(n\right)}\right)\right)\right]}^{-1}F^{\prime }\left(x^{\left(n\right)}\right)=I+2C_{2}\epsilon^{\left(n\right)}+\left(-2C_{2}^{2}+3C_{3}\right){\epsilon^{\left(n\right)}}^{2}+\left(-4C_{2}C_{3}+4C_{4}\right){\epsilon^{\left(n\right)}}^{3}\\ \; & +\left(5C_{5}+2C_{2}^{2}C_{3}-2C_{2}C_{3}C_{2}-3C_{3}C_{2}^{2}+4C_{2}^{4}-6C_{2}C_{4}\right){\epsilon^{\left(n\right)}}^{4}+O\left({\epsilon^{\left(n\right)}}^{5}\right). \end{array}$$

Then

(20) $$\begin{array}{ccc} H_{1}\left(x^{\left(n\right)}\right) & = & I+2C_{2}\epsilon^{\left(n\right)}-\left(C_{2}^{2}-3C_{3}\right){\epsilon^{\left(n\right)}}^{2}+\left(-C_{2}C_{3}-2C_{2}^{3}+4C_{4}\right){\epsilon^{\left(n\right)}}^{3}+O\left({\epsilon^{\left(n\right)}}^{4}\right). \end{array}$$

Using (14) and (17), we have

(21) $$\begin{array}{cc} \; & {\left[F^{\prime }\left(x^{\left(n\right)}\right)\right]}^{-1}F\left(y\left(x^{\left(n\right)}\right)\right)=C_{2}{\epsilon^{\left(n\right)}}^{2}+\left(2C_{3}-4C_{2}^{2}\right){\epsilon^{\left(n\right)}}^{3}+\left(13C_{2}^{3}-8C_{2}C_{3}-6C_{3}C_{2}+3C_{4}\right){\epsilon^{\left(n\right)}}^{4}\\ \; & +\left(-6C_{3}^{2}-10C_{2}^{4}+8C_{2}^{2}C_{3}+7C_{2}C_{3}C_{2}+6C_{3}C_{2}^{2}-6C_{2}C_{4}-4C_{4}C_{2}+4C_{5}\right){\epsilon^{\left(n\right)}}^{5}+O\left({\epsilon^{\left(n\right)}}^{6}\right). \end{array}$$

Then

(22) $$\begin{array}{c} \begin{array}{cc} & H_{1}\left(x^{\left(n\right)}\right){\left[F^{\prime }\left(x^{\left(n\right)}\right)\right]}^{-1}F\left(y\left(x^{\left(n\right)}\right)\right)=C_{2}{\epsilon^{\left(n\right)}}^{2}+\left(2C_{3}-2C_{2}^{2}\right){\epsilon^{\left(n\right)}}^{3}+\left(3C_{4}+4C_{2}^{3}-4C_{2}C_{3}-3C_{3}C_{2}\right){\epsilon^{\left(n\right)}}^{4}\\ & +\left(-6C_{3}^{2}+4C_{5}-6C_{2}C_{4}-4C_{4}C_{2}-22C_{2}^{4}+\frac{11}{2}C_{2}C_{3}C_{2}+8C_{2}^{2}C_{3}+\frac{39}{2}C_{3}C_{2}^{2}-12C_{2}^{2}C_{3}\right){\epsilon^{\left(n\right)}}^{5}+O\left({\epsilon^{\left(n\right)}}^{6}\right). \end{array} \end{array}$$

Using (16) and (22) in (9), thus the error is calculated as

$$\epsilon^{\left(n+1\right)}=\left(2C_{2}^{4}-C_{2}C_{3}C_{2}\right){\epsilon^{\left(n\right)}}^{5}+O\left({\epsilon^{\left(n\right)}}^{6}\right).$$

Thus, the above equation conform the fifth-order convergence. □

**Theorem** **2.** Let F be sufficiently differentiable in an open convex set D, $x^{\left(*\right)}\in D$, the roots of the problem F(x)=0. $F^{\prime }\left(x\right)$ is continuing function and nonsingular in $x^{\left(*\right)}$. Then the method (10) converging to the $x^{\left(*\right)}$ with convergence order at least 5+3r.

**Proof.**  A Taylor expansion of $F(\psi_{m-1}\left(x^{\left(n\right)}\right))$ around $x^{\left(*\right)}$ yields

(23) $$F\left(\psi_{m-1}\left(x^{\left(n\right)}\right)\right)=F^{\prime }\left(x^{\left(*\right)}\right)\left[\left(\psi_{m-1}\left(x^{\left(n\right)}\right)-x^{\left(*\right)}\right)+C_{2}{\left(\psi_{m-1}\left(x^{\left(n\right)}\right)-x^{\left(*\right)}\right)}^{2}+...\right]$$

In addition, let

(24) $$\begin{array}{ccc} H_{2}\left(x^{\left(n\right)}\right) & = & I+2C_{2}\epsilon^{\left(n\right)}+3C_{3}{\epsilon^{\left(n\right)}}^{2}+\left(2C_{2}C_{3}-4C_{2}^{3}+4C_{4}\right){\epsilon^{\left(n\right)}}^{3}+... \end{array}$$

Using (14) and (24), we have

(25) $$H_{2}\left(x^{\left(n\right)}\right)F{\left(x^{\left(n\right)}\right)}^{-1}=\left[I+M_{2}\;{\epsilon^{\left(n\right)}}^{3}+...\right]{\left[F^{\prime }\left(x^{\left(*\right)}\right)\right]}^{-1},\;M_{2}=2C_{2}C_{3}-4C_{2}^{3}$$

Using (23) and (25), we obtain

(26) $$\begin{array}{cc} \; & \psi_{m}\left(x^{\left(n\right)}\right)-x^{\left(*\right)}=\psi_{m-1}\left(x^{\left(n\right)}\right)-x^{\left(*\right)}-H_{2}\left(x^{\left(n\right)}\right)F{\left(x^{\left(n\right)}\right)}^{-1}F\left(\psi_{m-1}\left(x^{\left(n\right)}\right)\right)\\ \; & =\psi_{m-1}\left(x^{\left(n\right)}\right)-x^{\left(*\right)}-\left[I+M_{2}\;{\epsilon^{\left(n\right)}}^{3}+...\right]\;\left[\left(\psi_{m-1}\left(x^{\left(n\right)}\right)-x^{\left(*\right)}\right)+C_{2}{\left(\psi_{m-1}\left(x^{\left(n\right)}\right)-x^{\left(*\right)}\right)}^{2}+...\right]\\ \; & =-M_{2}\;{\epsilon^{\left(n\right)}}^{3}\left(\psi_{m-1}\left(x^{\left(n\right)}\right)-x^{\left(*\right)}\right)+... \end{array}$$

Proceeding by mathematical induction of (26) and using (11), we obtain

$$\psi_{r}\left(x^{\left(n\right)}\right)-x^{\left(*\right)}=M_{1}{\left(-M_{2}\right)}^{r}\;\left({\epsilon^{\left(n\right)}}^{\left(5+3r\right)}\right)+O\left({\epsilon^{\left(n\right)}}^{\left(3r+6\right)}\right),r\;\geq 1.$$

Thus, the proposed method conform that 5+3r order of convergence. □

## 3. Numerical Examples

In this section, numerical results are carried out using Matlab software. The contribute methods are used to approximate the solution of some nonlinear system of equations and they are compared with the results obtained for some existing methods.

(27) $$err_{min}={∥x^{\left(n+1\right)}-x^{\left(n\right)}∥}_{2}<10^{-100}.$$

In addition, approximated computational order of convergence $p_{c}$ is used, see ([18])

(28) $$p_{c}\approx \frac{\log\left(∥x^{\left(n+1\right)}-x^{\left(n\right)}{∥}_{2}/∥x^{\left(n\right)}-x^{\left(n-1\right)}{∥}_{2}\right)}{\log\left(∥x^{\left(n\right)}-x^{\left(n-1\right)}{∥}_{2}/∥x^{\left(n-1\right)}-x^{\left(n-2\right)}{∥}_{2}\right)}.$$

The following test problems are considered: **Test Problem 1** (TP1) (see [9])

$$F\left(x\right)=\left\{\begin{array}{c} x_{1}+exp\left(x_{2}\right)-cos\left(x_{2}\right)=0,\\ 3x_{1}-x_{2}-sin\left(x_{2}\right)=0. \end{array}\right.$$

Here initial points are $x^{\left(0\right)}={\left(1.5,2\right)}^{T}$, and the solution is $x^{\left(*\right)}={\left(0,0\right)}^{T}$.

**Test Problem 2** (TP2) (see [9])

$$F\left(x\right)=\left\{\begin{array}{c} x_{2}x_{3}+x_{4}\left(x_{2}+x_{3}\right)=0,\\ x_{1}x_{3}+x_{4}\left(x_{1}+x_{3}\right)=0,\\ x_{1}x_{2}+x_{4}\left(x_{1}+x_{2}\right)=0,\\ x_{1}x_{2}+x_{1}x_{3}+x_{2}x_{3}=1. \end{array}\right.$$

Here initial points are $x^{\left(0\right)}={\left(0.5,0.5,0.5,-0.2\right)}^{T}$. 

The solution is $x^{\left(*\right)}\approx {\left(0.577350,0.577350,0.577350,-0.288675\right)}^{T}$.

**Test Problem 3** (TP3) (see [9])

$$F\left(x\right)=\left\{\begin{array}{c} x_{i}x_{i+1}-1=0,\;\;i=1,2,...15,\\ x_{15}x_{1}-1=0. \end{array}\right.$$

Here $x^{\left(0\right)}={\left(1.5,1.5,1.5,...,1.5\right)}^{T}$, and $x^{\left(*\right)}={\left(1,1,1,...,1\right)}^{T}$.

Table 1, shows the results for the above test problems, where *M* represents the number iterations required for convergence. Here the conventional method $2^{nd}NR$ is converging with 10, 8, and 9 iterations for TP1,TP2 and TP3 respectively, whereas the proposed method $11^{th}PM,r=2$ is converging with 4, 3, and 4 iterations for TP1,TP2 and TP3 respectively. All other compared methods are converging slower than the new $11^{th}PM,r=2$ method. Hence, we conclude that $11^{th}PM,r=2$ method is the efficient one compared with other methods.

## 4. Efficiency of the Methods

We utilize the efficiency index (EI), $EI=p^{\frac{1}{d}}$ ([1]), *p* represent that the order, *d* represent the total functional evaluations. This is the foremost utilized record, and another one we utilize is the computational efficiency index (CE) characterized as $CE=p^{1/(d+op)}$ ([5]), where op is the operations cost per cycle. We review that the number of items and remainders that to be solved using LU factorization ($\frac{1}{3}n^{3}+mn^{2}-\frac{1}{3}n$), where *n* is the system size for *m* linear systems with the same matrix of the coefficient.

**Figure 2:**
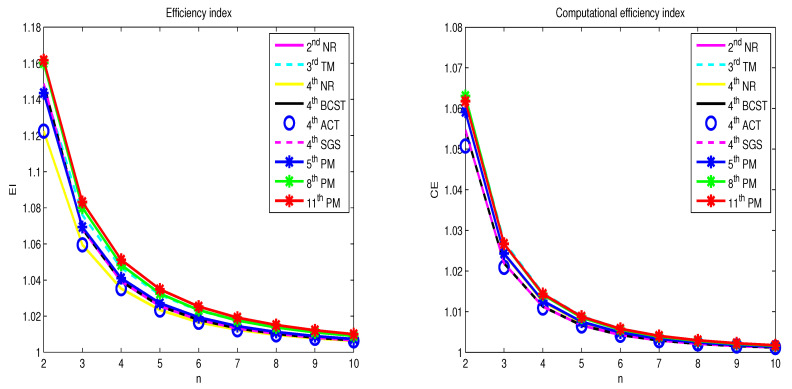
Comparison of EI and CE.

**Figure 3:**
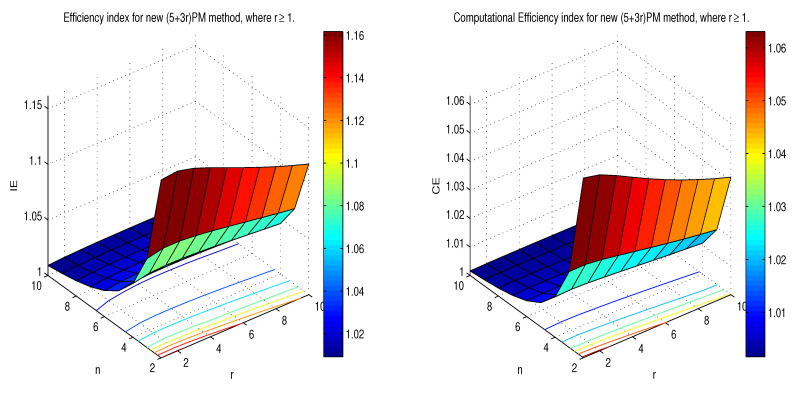
Comparison of EI and CE for (5+3r)PM method, where r≥1.

Table 2 gives the result of EI and CE expression for the methods discussed above. Figure 2 shows the execution of diverse methods with regard to EI and CE. It is identified that the new methods perform better than other methods for n≥2. Figure 3 displays the performance of the proposed (5+3r)PM method with respect to EI and CE where r≥1. When the size of system *n* and step size *r* increase then the EI and CE decrease respectively, we noted here, that new methods yield good EI and CE with n≤6 and r≤3.

## 5. Applications on Global Positioning System (GPS)

### 5.1. Basics on GPS

The worldwide coverage of all satellites has been provided by the space-based navigation systems like GPS around the clock to the users. There are minimum of 24 satellites positioned in a circular orbital constellation with an approximate altitude of 20,000 km above from the earth surface. To solve the user position from the satellite constellation, the nonlinear pseuorange equations need to be solved with higher precision. The methods like linearization and point iteration technique are used to solve the pseudorange equations. Actually, the solutions for these nonlinear equations are in the Cartesian coordinate system, especially in the ECEF format. However, the Earth is not in the shape of a perfect sphere, therefore, once the position of the user is calculated, the coordinates have to be changed into a spherical system that is suitable for latitude, longitude, and altitude form at to integrate the user position in to a MAP application. The position of an unknown point (user position) in the space can be determined by measuring the distance from the known position in space i.e., satellite position to the unknown user position.

**Figure 4:**
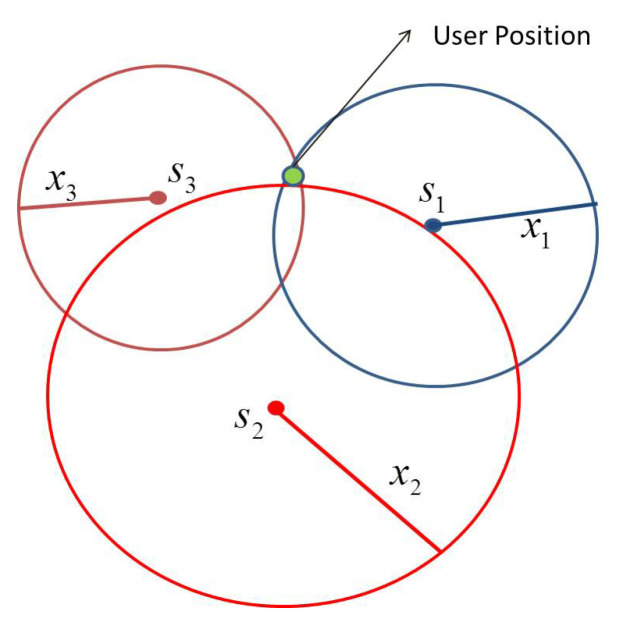
Two dimensional user position.

In the two-dimensional case of user position determination, as shown in Figure 4, the three distances $x_{sat1},x_{sat2},x_{sat3}$ are required from the three satellites ($s_{1},s_{2}$ and $s_{3}$) center point of the trace of a circle. Two possible solutions can be obtained from two satellites with two distances that create at two points the two circles together will intersect.

**Figure 5:**
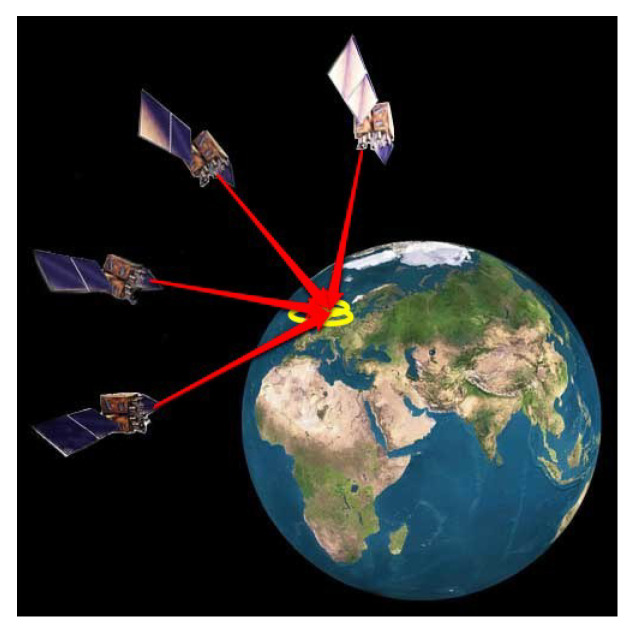
Three dimensional user position ([20]).

However, from this the user position cannot be determined uniquely. Therefore, one can use four satellites with four distances to proceed with a three-dimensional case. As shown in Figure 5, in a three-dimensional case, the centred point in an earth sphere forms an equal-distance trace. The transmitted ephemeris data of the satellite information gives the orbital information from this, one can determine the distance of the satellite easily. For more information refer to the literatures [19].

### 5.2. Measurement of Pseudorange

The common bias is known as pseudorange which is measured by a receiver from the ranges to GPS satellites. The distance between the user and the satellite geometric range multiplied by the velocity of light (c) is known as pseudorange ($\rho_{i}$) that is the difference in time required between the transmitted and the received signal which is calculated on the L1 frequency signal is written as (see [19])

(29) $$\rho_{i}=R_{i}+c.\Delta t-d_{sat}+d_{iono}+d_{tropo}+d_{rel}+d_{ins}$$

where $R_{i}$—Geometric range,

c.Δt—Unknown distance caused by the receiver clock offset,
$d_{sat}$ —Advance of the satellite clock with respect to system time,
$d_{iono}$—Ionospheric delay,
$d_{tropo}$—Tropospheric delay,
$d_{rel}$—Relativistic delay, and
$d_{ins}$—Instrumental delay.

The various correction models need to be used to minimize the errors, however, in this case, we assumed that the errors are negligible to some extent. In this case, the pseudorange equations can be given as

(30) $$\rho_{i}=R_{i}+c.\Delta t.$$

The geometric range can be written as

(31) $$R_{i}=\sqrt{{\left(x_{sati}-x_{user}\right)}^{2}+{\left(y_{sati}-y_{user}\right)}^{2}+{\left(z_{sati}-z_{user}\right)}^{2}}.$$

where $\rho_{i},x_{sati},y_{sati},z_{sati}$ are known and $x_{user},y_{user},z_{user},\Delta t$ are unknown, a minimum of four satellites coordinates are enough to find out the solution that is the user position values for i=1,2,3,4 as given by Equation (4) (see [19])

(32) $$\begin{array}{cc} \; & \rho_{1}=\sqrt{{\left(x_{sat1}-x_{user}\right)}^{2}+{\left(y_{sat1}-y_{user}\right)}^{2}+{\left(z_{sat1}-z_{user}\right)}^{2}}+c\Delta t,\\ \; & \rho_{2}=\sqrt{{\left(x_{sat2}-x_{user}\right)}^{2}+{\left(y_{sat2}-y_{user}\right)}^{2}+{\left(z_{sat2}-z_{user}\right)}^{2}}+c\Delta t,\\ \; & \rho_{3}=\sqrt{{\left(x_{sat3}-x_{user}\right)}^{2}+{\left(y_{sat3}-y_{user}\right)}^{2}+{\left(z_{sat3}-z_{user}\right)}^{2}}+c\Delta t,\\ \; & \rho_{4}=\sqrt{{\left(x_{sat4}-x_{user}\right)}^{2}+{\left(y_{sat4}-y_{user}\right)}^{2}+{\left(z_{sat4}-z_{user}\right)}^{2}}+c\Delta t,\\ \; & \vdots \\ \; & \rho_{n}=\sqrt{{\left(x_{satn}-x_{user}\right)}^{2}+{\left(y_{satn}-y_{user}\right)}^{2}+{\left(z_{satn}-z_{user}\right)}^{2}}+c\Delta t. \end{array}$$

### 5.3. Solving Nonlinear Pseudorange Equations

In simplified form, the pseudorange equations can be written in general for solving the above system of equations as

(33) $$\begin{array}{cc} \; & \rho_{i}=\sqrt{{\left(x_{sati}-x_{user}\right)}^{2}+{\left(y_{sati}-y_{user}\right)}^{2}+{\left(z_{sati}-z_{user}\right)}^{2}}+b_{user},\;\;\;i=1\left(1\right)n \end{array}$$

where $b_{user}$ is the user clock bias error expressed in distance, by differentiating the above equation, we have

(34) $$\begin{array}{cc} \; & \delta \rho_{i}=\frac{\left(x_{sati}-x_{user}\right)\delta x_{user}+\left(y_{sati}-y_{user}\right)\delta y_{user}+\left(z_{sati}-z_{user}\right)\delta z_{user}}{\rho_{i}-b_{user}}+\delta b_{user}. \end{array}$$

The variables $x_{user},\;y_{user},\;z_{user},\;b_{user}$ are termed as known quantities and the initialization values of these variables can be assumed as center of the earth. A new set of values $\delta x_{user},\;\delta y_{user},\;\delta z_{user},\;\delta b_{user}$ can be calculated from the initial values. The new set of values calculated from the previous step are utilized to add with the present values $x_{user},\;y_{user},\;z_{user},\;b_{user}$ for finding next new set of solutions. To get a desired solution as the final value of $\delta x_{user},\;\delta y_{user},\;\delta z_{user},\;\delta b_{user}$, the procedure is continued until the absolute values of $\delta x_{user},\;\delta y_{user},\;\delta z_{user},\;\delta b_{user}$ are obtained very small within the predefined values. The above procedure is generally known as linearization of iteration method of fixed point. The expression of above equation becomes a set of linear equations that can be written in matrix form as (see [19])

(35) $$\left(\begin{array}{c} \delta \rho_{1}\\ \delta \rho_{2}\\ \delta \rho_{3}\\ \delta \rho_{4} \end{array}\right)=\left(\begin{array}{cccc} \alpha_{11} & \alpha_{12} & \alpha_{13} & 1\\ \alpha_{21} & \alpha_{22} & \alpha_{23} & 1\\ \alpha_{31} & \alpha_{32} & \alpha_{33} & 1\\ \alpha_{41} & \alpha_{42} & \alpha_{43} & 1 \end{array}\right)\left(\begin{array}{c} \delta x_{user}\\ \delta y_{user}\\ \delta z_{user}\\ \delta b_{user} \end{array}\right),$$

where

(36) $$\alpha_{i1}=\frac{x_{sati}-x_{user}}{\rho_{i}-b_{user}},\;\alpha_{i2}=\frac{y_{sati}-y_{user}}{\rho_{i}-b_{user}},\;\alpha_{i3}=\frac{z_{sati}-z_{user}}{\rho_{i}-b_{user}}.$$

The solution of (35) is

(37) $$\left(\begin{array}{c} \delta x_{user}\\ \delta y_{user}\\ \delta z_{user}\\ \delta b_{user} \end{array}\right)={\left(\begin{array}{cccc} \alpha_{11} & \alpha_{12} & \alpha_{13} & 1\\ \alpha_{21} & \alpha_{22} & \alpha_{23} & 1\\ \alpha_{31} & \alpha_{32} & \alpha_{33} & 1\\ \alpha_{41} & \alpha_{42} & \alpha_{43} & 1 \end{array}\right)}^{-1}\left(\begin{array}{c} \delta \rho_{1}\\ \delta \rho_{2}\\ \delta \rho_{3}\\ \delta \rho_{4} \end{array}\right).$$

This process noticeably does not give the required solutions straightforwardly. In any case, the desired results can be obtained from it. In order to determine the required user position solution, an iterative way could be utilized repetitively to solve this technique. A measure is often used to determine whether the desired result is achieved, and this quantity can be defined as (see [19])

(38) $$\begin{array}{cc} \; & \delta v=\sqrt{\delta x_{user}^{2}+\delta y_{user}^{2}+\delta z_{user}^{2}+\delta b_{user}^{2}}, \end{array}$$

where δv is lower than a certain predetermined threshold, the iteration will stop. Sometimes, the clock bias $b_{user}$ is not included in (38). In this work, we use the norm $∥x^{\left(k+1\right)}-x^{\left(k\right)}{∥}_{2}$ for the stopping criterion because it is stronger than (38). In this GNSS problem, we set the stopping criteria in such a way that the error $err_{min}={∥x^{\left(k+1\right)}-x^{\left(k\right)}∥}_{2}<10^{-10}$ should be less than the predefined value within the number of iterations(‘M’) required for reaching the minimum residual value.

## 6. Results and Discussion

The test problems are solved using the above-mentioned iterative methods. The second order Newton method ($2^{nd}NR$) takes longer iteration to converge the error when compared to all methods. The methods like $3^{rd}TM$, $4^{th}NR$, $4^{th}BCST$, $4^{th}ACT$, and $4^{th}SGS$ converge within five or six iterations to solve the test problems with lower bound error. Finally testing with proposed ($5^{th}PM,8^{th}PM,11^{th}PM$) methods achieved the solution within 4 iterations. So, this could be the optimal choice for solving non-linear equations.

**Figure 6:**
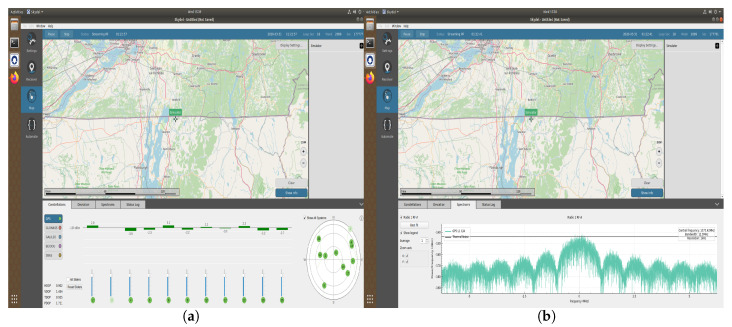
(**a**) Coordinates used in simulation (Map with skyplot). (**b**) Coordinates used in simulation (Map with spectrum of GPS signal).

Next, the iterative methods are tested for solving GPS pseudo-range equations. The satellite coordinates and the pseudorange information are collected from the OroIia GNSS simulator in which the location has been set as ETS, Montreal as shown in Figure 6.

To carry out the problem of finding the user position, the following coordinates of satellites and its pseudorange values are obtained from the Orolia GNSS simulator workbench installed in LASSENA lab is given in Table 3.

In Table 4, Table 5 and Table 6 we compare the proposed method with other iterative methods used in GPS receiver, where *M* represents the number iterations required for convergence. We recall that the coordinates of the center of the Earth and $b_{user}=0$ gives $x^{\left(0\right)}={\left(0,0,0\right)}^{T}$ is usually used as starting value. We denote $x^{\left(*\right)}={\left(1266370.3895,-4279711.6757,4446203.8082\right)}^{T}$ as the solution of the nonlinear system which gives the user position on the Earth. Thus, we conclude that $5^{th}PM$ method is the most efficient method compared to other methods. In general, our proposed scheme converges in lesser iterations than other tested methods with least error and less cpu time. The results indicate that higher-order multi-point iterative methods are simple, fast, and more efficient than other compared methods.

In addition, the initial points $x^{\left(0\right)}={\left(10^{6},10^{6},10^{6}\right)}^{T}$ and $x^{\left(0\right)}={\left(-10^{4},-10^{4},-10^{4}\right)}^{T}$ are used here to find the user position. For the first initial point, the user position is found exterior of the space solution, that is $x^{\left(d\right)}={\left(785566.9552,8099956.9218,-19562198.7778\right)}^{T}$. For the second point, it converges to the user position with six iterations whereas centre of the earth point converges within three iterations. Hence, centre of earth is always efficient one to use as initial point to find the user position.

**Figure 7:**
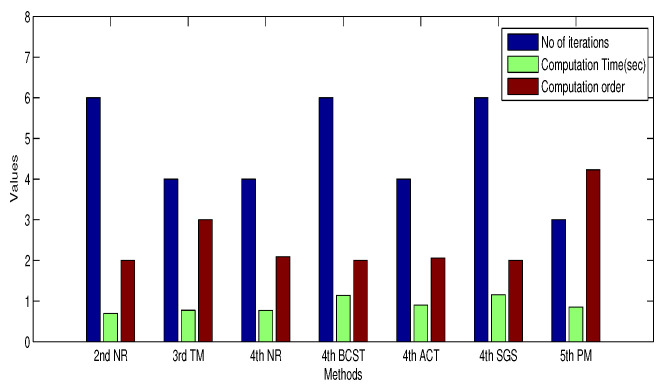
Comparison of various iterative methods.

The available satellites are kept as eight; out of these, initially, we have considered only four satellites for finding the user position. From the comparison results plotted in Figure 7, it has been observed that the 5th order PM method converged quickly and took les computation with fewer iterations when compared to the other methods. Similarly, the error is also computed for all other iterative methods, while finding the solution for test problems, the 5^th^ order, 8th order and 11th order PM methods almost converged within fourth or fifth iterations with lesser computation time; that is the reason we have chosen only 5^th^*PM* only and later two higher order methods are not included in solving GPS pseudo range equations.

We increased the satellites count from four to eight. The GDOP value is computed for three cases, among all the methods, 5th order proposed method converges within three iterations with least error of $1.6324\times 10^{-12}$. The Fuzzy 2 based satellite selection algorithm [21] is used here for selecting the best ‘n’ combinations of visible satellites. The GDOP value remains same as 0.40563 after increasing the satellites count beyond six. Moreover, for different inputs of all visible satellites, the user position is computed. For instance, from 6 to 8 satellites are considered for computing the user position, the family of iterative methods yield very good performance in terms of lesser iterations and computation time when compared to the traditional Taylor series and Bancraft methods.

The comparison of position fix error for iterative method mentioned in the open source [22] and the proposed method for each iteration is calculated as shown in Table 7, Table 8 and Table 9 and it has been observed that the error in each iteration for four, five and six satellite scenarios, the proposed method converged quickly within three iterations.

## 7. Concluding Remarks

In the foregoing article, we have analyzed the order of convergence and numerical results of new two-step fifth and multi-step iterative methods. Consequently, we are getting a better results than other existing discussed methods. The most useful benefit of the new scheme as they don’t utilize second order Frechet derivative. For practical applications, it is found that all the iterative methods converge to the user position measured from the center of the earth. Among the methods tested, 5th PM method is converged to the user position with a smaller number of iterations and having less CPU time and error for solving both numerical problems and GNSS pseudorange equations. In the future, it is possible implement the position calculation module with multi constellation GNSS receivers that employ GPS, GLONASS, GALELIO, BEIDOU, QZSS and IRNSS satellite navigation systems. At present more than 70 satellites are already in view and once all major four navigation systems (BeiDou + Galileo + GLONASS + GPS) are put in to orbit then more than 120 satellites will be available to the users. At any point of time an average around 30 satellites are visible from most locations around the world. If any one of the navigation systems fails then we rely on other satellite navigation systems. The recently developed GNSS receivers, a maximum of 54 channels can be allotted to different satellite navigation systems. The number of visible satellites can be chosen from the signals of opportunity then one can select more than four visible satellites (mixed GNSS Satellites) for computing better GDOP values and thus by using our proposed higher-order multi-point iterative methods, the user position can be determined more accurately with a lesser number of iterations in lower computation time.

## Figures and Tables

**Table 1:** Numerical results on different iterative methods.

Methods	TP1			TP2			TP3		
	M	${\mathrm{err}}_{\min}$	$p_{c}$	M	${\mathrm{err}}_{\min}$	$p_{c}$	M	${\mathrm{err}}_{\min}$	$p_{c}$
$2^{nd}NR$	10	$1.03\times 10^{-103}$	1.99	8	$3.92\times 10^{-145}$	2.00	9	$8.96\times 10^{-179}$	1.99
$3^{rd}TM$	7	$9.65\times 10^{-104}$	2.99	6	$8.87\times 10^{-236}$	3.01	6	$5.79\times 10^{-142}$	2.99
$4^{th}NR$	6	$5.38\times 10^{-207}$	3.99	5	$2.98\times 10^{-291}$	4.03	5	$8.96\times 10^{-179}$	3.99
$4^{th}BCST$	6	$5.05\times 10^{-139}$	3.99	5	$3.49\times 10^{-238}$	4.03	5	$1.61\times 10^{-142}$	3.99
$4^{th}ACT$	6	$2.80\times 10^{-309}$	3.99	5	$3.86\times 10^{-283}$	4.03	5	$2.03\times 10^{-203}$	3.99
$4^{th}SGS$	6	$2.22\times 10^{-170}$	3.99	5	$8.89\times 10^{-257}$	4.03	5	$6.08\times 10^{-155}$	3.99
$5^{th}PM,r=0$	5	$2.41\times 10^{-114}$	4.85	4	$1.46\times 10^{-131}$	5.30	5	$0.00\times 10^{-0}$	4.99
$8^{th}PM,r=1$	5	$0.00\times 10^{-0}$	7.99	4	$0.00\times 10^{-0}$	7.99	4	$1.60\times 10^{-301}$	7.99
$11^{th}PM,r=2$	4	$2.37\times 10^{-191}$	10.96	3	$1.89\times 10^{-118}$	11.65	4	$0.00\times 10^{-0}$	10.99

**Table 2:** Comparison of EI and CE.

Method	EI	CE
$2^{nd}NR$	$2^{\frac{1}{n+n^{2}}}$	$2^{\frac{1}{\frac{1}{3}n^{3}+2n^{2}+\frac{2}{3}n}}$
$3^{rd}TM$	$3^{\frac{1}{2n+n^{2}}}$	$3^{\frac{1}{\frac{1}{3}n^{3}+3n^{2}+\frac{5}{3}n}}$
$4^{th}NR$	$4^{\frac{1}{2n+2n^{2}}}$	$4^{\frac{1}{\frac{2}{3}n^{3}+4n^{2}+\frac{4}{3}n}}$
$4^{th}BCST$	$4^{\frac{1}{n+2n^{2}}}$	$4^{\frac{1}{\frac{2}{3}n^{3}+5n^{2}+\frac{1}{3}n}}$
$4^{th}ACT$	$4^{\frac{1}{2n+2n^{2}}}$	$4^{\frac{1}{\frac{2}{3}n^{3}+5n^{2}+\frac{4}{3}n}}$
$4^{th}SGS$	$4^{\frac{1}{n+2n^{2}}}$	$4^{\frac{1}{\frac{2}{3}n^{3}+5n^{2}+\frac{4}{3}n}}$
$5^{th}PM$	$5^{\frac{1}{2n+2n^{2}}}$	$5^{\frac{1}{\frac{2}{3}n^{3}+5n^{2}+\frac{4}{3}n}}$
$8^{th}PM$	$8^{\frac{1}{3n+2n^{2}}}$	$8^{\frac{1}{\frac{2}{3}n^{3}+6n^{2}+\frac{7}{3}n}}$
$11^{th}PM$	$11^{\frac{1}{4n+2n^{2}}}$	$11^{\frac{1}{\frac{2}{3}n^{3}+7n^{2}+\frac{10}{3}n}}$

**Table 3:** Coordinates of observed satellite and pseudorange.

*X*	*Y*	*Z*	ρ
5,731,058.70224	14,489,833.1300	21,212,629.60495	20,626,320.6953
14,647,763.94672	6,275,922.21243	21,269,863.31368	22,047,954.0019
2,505,594.09552	20,672,767.43360	16,516,258.99994	21,238,160.1101
21,359,664.8576	12,371,174.84498	10,076,109.65011	252,188.013893
15,520,711.16562	1,047,695.53474	21,458,920.6173	23,317,558.30921
12,374,316.48857	23,533,071.9923	1,008,701.68425	23,125,967.55620
14,253,263.56829	4,103,289.05642	21,789,614.16879	23,747,495.1073
15,759,895.08320	5,849,736.59321	20,805,729.39674	24,788,995.2855

**Table 4:** Comparison of iterative methods for GPS problem with 4 satellites scenario.

Method	*M*	$∥x^{\left(k-1\right)}-x^{\left(k-2\right)}{∥}_{2}$	$∥x^{\left(k\right)}-x^{\left(k-1\right)}{∥}_{2}$	$∥x^{\left(k+1\right)}-x^{\left(k\right)}{∥}_{2}$	CPU(sec)	$p_{c}$	GDOP
$2^{nd}NR$	6	$0.2271\times 10^{0}$	$1.9087\times 10^{-9}$	$1.3480\times 10^{-25}$	0.695	2.00	
$3^{rd}TM$	4	2.6206 $\times 10^{4}$	$0.0495\times 10^{0}$	$3.3212\times 10^{-19}$	0.774	3.00	
$4^{th}NR$	4	2.4777 $\times 10^{3}$	1.9087 $\times 10^{-9}$	2.4095 $\times 10^{-32}$	0.772	2.09	
$4^{th}BCST$	6	$21.1858\times 10^{0}$	3.3216$\times 10^{-5}$	8.1651 $\times 10^{-17}$	1.137	2.00	0.5265
$4^{th}ACT$	4	1.9374$\times 10^{3}$	7.1420 $\times 10^{-10}$	2.7733 $\times 10^{-32}$	0.903	2.06	
$4^{th}SGS$	6	$21.3566\times 10^{0}$	3.3754 $\times 10^{-5}$	8.4317 $\times 10^{-17}$	1.157	2.00	
$5^{th}PM$	3	6.2946 $\times 10^{6}$	3.3716 $\times 10^{3}$	1.6324 $\times 10^{-12}$	0.853	4.23	

**Table 5:** Comparison of iterative methods for GPS problem with 5 satellites scenario.

Method	*M*	$∥x^{\left(k-1\right)}-x^{\left(k-2\right)}{∥}_{2}$	$∥x^{\left(k\right)}-x^{\left(k-1\right)}{∥}_{2}$	$∥x^{\left(k+1\right)}-x^{\left(k\right)}{∥}_{2}$	CPU(sec)	$p_{c}$	GDOP
$2^{nd}NR$	6	$0.2577\times 10^{0}$	$2.8677\times 10^{-6}$	1.3444$\times 10^{-11}$	0.795	2.00	
$3^{rd}TM$	5	$0.3489\times 10^{0}$	2.0554$\times 10^{-6}$	9.6836$\times 10^{-12}$	0.974	2.96	
$4^{th}NR$	4	2.5209$\times 10^{3}$	2.8677 $\times 10^{-6}$	$6.3505\times 10^{-17}$	0.838	2.27	
$4^{th}BCST$	7	$3.6589\times 10^{-4}$	$2.6656\times 10^{-9}$	$1.6758\times 10^{-14}$	1.406	2.00	0.4654
$4^{th}ACT$	4	1.9676$\times 10^{3}$	$1.0730\times 10^{-7}$	$2.4238\times 10^{-18}$	1.014	2.12	
$4^{th}SGS$	7	$2.8345\times 10^{-4}$	$1.6174\times 10^{-9}$	$7.6224\times 10^{-15}$	1.460	2.00	
$5^{th}PM$	3	$6.3604\times 10^{6}$	$2.6147\times 10^{3}$	$1.2351\times 10^{-11}$	0.754	4.23	

**Table 6:** Comparison of iterative methods for GPS problem with 6 satellites scenario.

Method	*M*	$∥x^{\left(k-1\right)}-x^{\left(k-2\right)}{∥}_{2}$	$∥x^{\left(k\right)}-x^{\left(k-1\right)}{∥}_{2}$	$∥x^{\left(k+1\right)}-x^{\left(k\right)}{∥}_{2}$	CPU(sec)	$p_{c}$	GDOP
$2^{nd}NR$	6	$0.4495\times 10^{0}$	$1.4550\times 10^{-8}$	$1.4317\times 10^{-16}$	0.866	2.00	
$3^{rd}TM$	4	$7.8259\times 10^{3}$	$0.0064\times 10^{0}$	$5.6447\times 10^{-11}$	0.957	2.85	
$4^{th}NR$	4	$2.3459\times 10^{3}$	$1.4550\times 10^{-8}$	$9.1394\times 10^{-26}$	0.954	2.23	
$4^{th}BCST$	6	$54.5307\times 10^{0}$	$4.8575\times 10^{-4}$	$5.6948\times 10^{-12}$	1.413	1.99	0.4075
$4^{th}ACT$	4	$2.0707\times 10^{3}$	$1.0053\times 10^{-8}$	$5.6676\times 10^{-26}$	1.107	2.10	
$4^{th}SGS$	6	$54.7063\times 10^{0}$	$4.8898\times 10^{-4}$	$4.2930\times 10^{-12}$	1.428	1.99	
$5^{th}PM$	3	$6.3604\times 10^{6}$	$2.5886\times 10^{3}$	$3.3911\times 10^{-11}$	0.831	4.09	

**Table 7:** Results of error calculation in 5^th^PM and GPS tool box [22] with four satellites scenario.

Iterative Method (GPS Tool Box)		Proposed Method (5^*th*^PM)	
M	**Error**	M	**Error**
1	$6.3675\times 10^{6}$	1	$6.2946\times 10^{6}$
2	$2.0978\times 10^{5}$	2	$3.3716\times 10^{3}$
3	$1.2709\times 10^{3}$	3	$1.6324\times 10^{-12}$
4	0.0460		
5	$2.8649\times 10^{-8}$		
6	$7.7721\times 10^{-8}$		
7	0		

**Table 8:** Results of error calculation in 5^th^PM and GPS tool box [22] with five satellites scenario.

Iterative Method (GPS Tool Box)		Proposed Method (5^*th*^PM)	
M	**Error**	M	**Error**
1	$1.3363\times 10^{6}$	1	$6.3604\times 10^{6}$
2	$6.7220\times 10^{4}$	2	$2.6147\times 10^{3}$
3	168.6654	3	$1.2351\times 10^{-11}$
4	0.0011		
5	$1.7198\times 10^{-8}$		
6	$1.4639\times 10^{-8}$		
7	$1.1727\times 10^{-8}$		
8	$1.4233\times 10^{-8}$		
9	$2.7819\times 10^{-8}$		
10	0		

**Table 9:** Results of error calculation in 5^th^PM and GPS tool box [22] with six satellites scenario.

Iterative Method (GPS Tool Box)		Proposed Method (5^*th*^PM)	
M	**Error**	M	**Error**
1	$1.9700\times 10^{6}$	1	$6.3604\times 10^{6}$
2	$1.1879\times 10^{4}$	2	$2.5886\times 10^{3}$
3	441.8016	3	$3.3911\times 10^{-11}$
4	0.0064		
5	$2.0352\times 10^{-8}$		
6	$1.0844\times 10^{-8}$		
7	$1.9246\times 10^{-8}$		
8	$9.4634\times 10^{-9}$		
9	$1.7549\times 10^{-8}$		
10	$3.0064\times 10^{-9}$		
11	$5.3703\times 10^{-8}$		
12	0		

## References

[1] Ostrowski A.M. (1960). Solutions of Equations and System of Equations.

[2] Traub J.F. (1964). Iterative Methods for the Solution of Equations.

[3] Abad M.F., Cordero A., Torregrosa J.R. (2013). Fourth- and Fifth-Order Methods for Solving Nonlinear Systems of Equations: An Application to the Global Positioning System. Abstr. Appl. Anal..

[4] Babajee D., Madhu K., Jayaraman J. (2015). On Some Improved Harmonic Mean Newton-Like Methods for Solving Systems of Nonlinear Equations. Algorithms.

[5] Cordero A., Hueso J.L., Martinez E., Torregrosa J.R. (2010). A modified Newton-Jarratt’s composition. Numer. Algor..

[6] Noor M.A., Waseem M., Noor K.I., Al-Said E. (2013). Variational iteration technique for solving a system of nonlinear equations. Optim. Lett..

[7] Sharma J.R., Guha R.K., Sharma R. (2013). An efficient fourth order weighted-Newton method for systems of nonlinear equations. Numer. Algor..

[8] Babajee D.K.R., Cordero A., Soleymani F., Torregrosa J.R. (2012). On a Novel Fourth-Order Algorithm for Solving Systems of Nonlinear Equations. J. Appl. Math..

[9] Madhu K., Babajee D., Jayaraman J. (2017). An improvement to double-step Newton method and its multi-step version for solving system of nonlinear equations and its applications. Numer. Algor..

[10] Yang M. (2005). Noniterative Method of Solving the GPS Double-Differenced Pseudorange Equations. J. Surv. Eng..

[11] Pachter M., Nguyen T.Q. (2003). An Efficient GPS Position Determination Algorithm. J. Inst. Navig..

[12] Li W., Yang S.H., Li D., Xu Y.W., Zhao W. Design and Analysis of a New GPS Algorithm. Proceedings of the 2010 IEEE 30th International Conference on Distributed Computing Systems.

[13] Ko K.S., Choi C.M. (2010). Mathematical Algorithms for Two-Dimensional Positioning Based on GPS Pseudorange Technique. Int. J. Kim..

[14] Jwoa D., Hsiehb M., Leea Y. (2015). GPS navigation solution using the iterative least absolute deviation approach. Sci. Iran. B.

[15] Awange J.L., Grafarend E.W. (2002). Algebraic Solution of GPS Pseudo-Ranging Equations. GPS Solut..

[16] El-naggar A.M. (2011). An alternative methodology for the mathematical treatment of GPS positioning. Alex. Eng. J..

[17] Cordero A., Gomez E., Torregrosa J.R. (2017). Efficient High-Order Iterative Methods for Solving Nonlinear Systems and Their Application on Heat Conduction Problems. Complexity.

[18] Cordero A., Torregrosa J.R. (2007). Variants of Newton’s method using fifth-order quadrature formulas. Appl. Math. Comp..

[19] Tsui J.B.Y. (2005). Fundamentals of Global Positioning System Receivers, a Software Approach.

[20] Griffin D. (2011). How does the Global Positioning System Work?. http://www.pocketgpsworld.com/howgpsworks.php.

[21] Arul-Elango G., Murukesh C., Rajeswari K. (2016). Type-2 Fuzzy based GPS Satellite Selection algorithm for better Geometrical Dilution of Precision. Int. J. Comput. Sci. Inf. Secur..

[22] Tetewsky A.K., Soltz A. (1998). GPS MATLAB Toolbox Review. GPS World.

